# Correction: The 20-minute whole blood clotting test (20WBCT) for snakebite coagulopathy—A systematic review and meta-analysis of diagnostic test accuracy

**DOI:** 10.1371/journal.pntd.0011080

**Published:** 2023-01-24

**Authors:** Thomas Lamb, Michael Abouyannis, Sâmella Silva de Oliveira, Rachana Shenoy K., Tulasi Geevar, Anand Zachariah, Sanjib Kumar Sharma, Navin Bhatt, Mavuto Mukaka, Eli Harriss, David G. Lalloo, Elizabeth A. Ashley, Wuelton Marcelo Monteiro, Frank Smithuis, Michael Eddleston

There are errors in Fig 5 and Fig 8. Fig 8 is in the position Fig 5 should be in and Fig 5 is in the position Fig 8 should be in. The correct Fig 5 and Fig 8 are provided.

**Fig 5:**
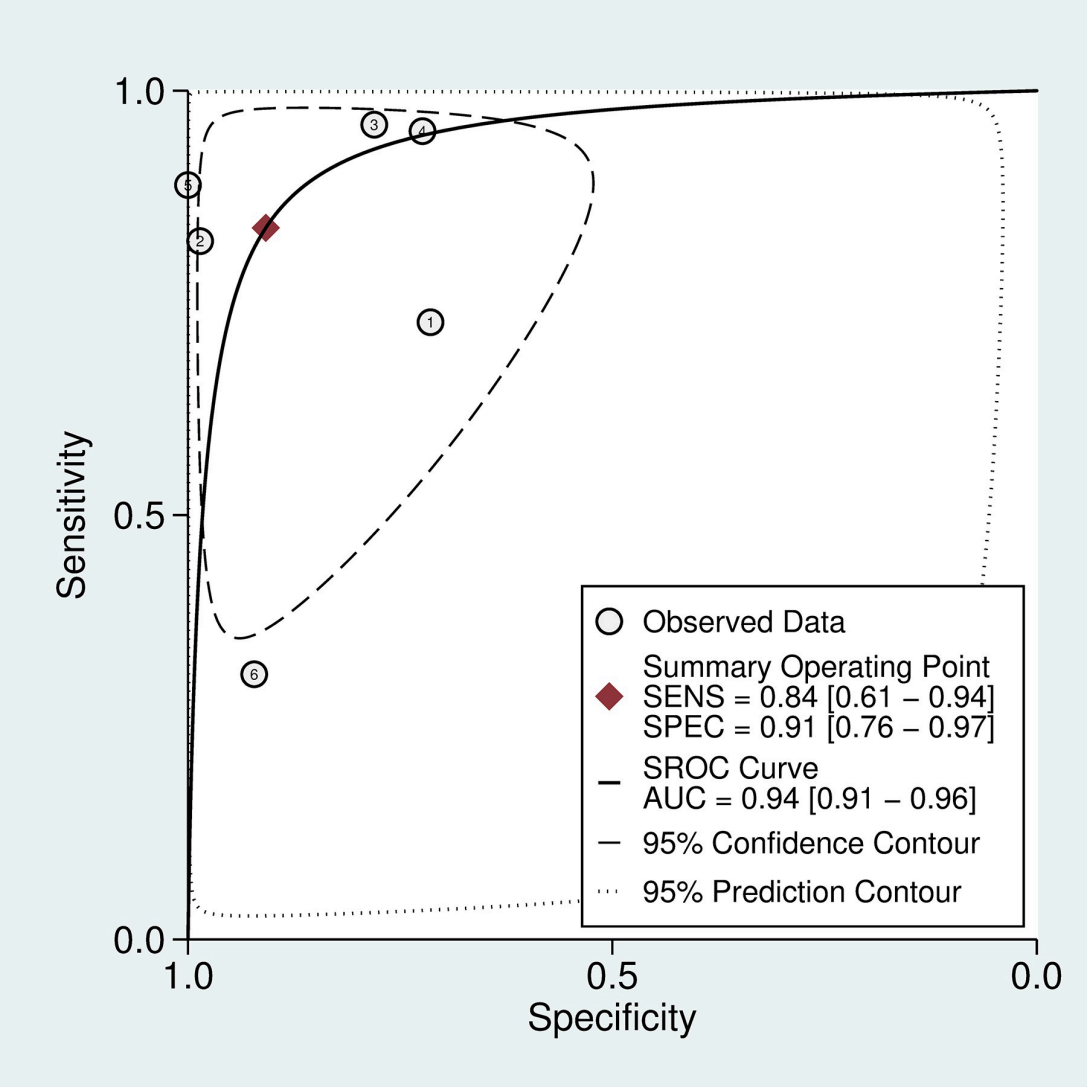
SROC curve for 20WBCT at detecting coagulopathy. SROC curves for 20WBCT at detecting mild coagulopathy defined as fibrinogen <100 mg/dL.

**Fig 8:**
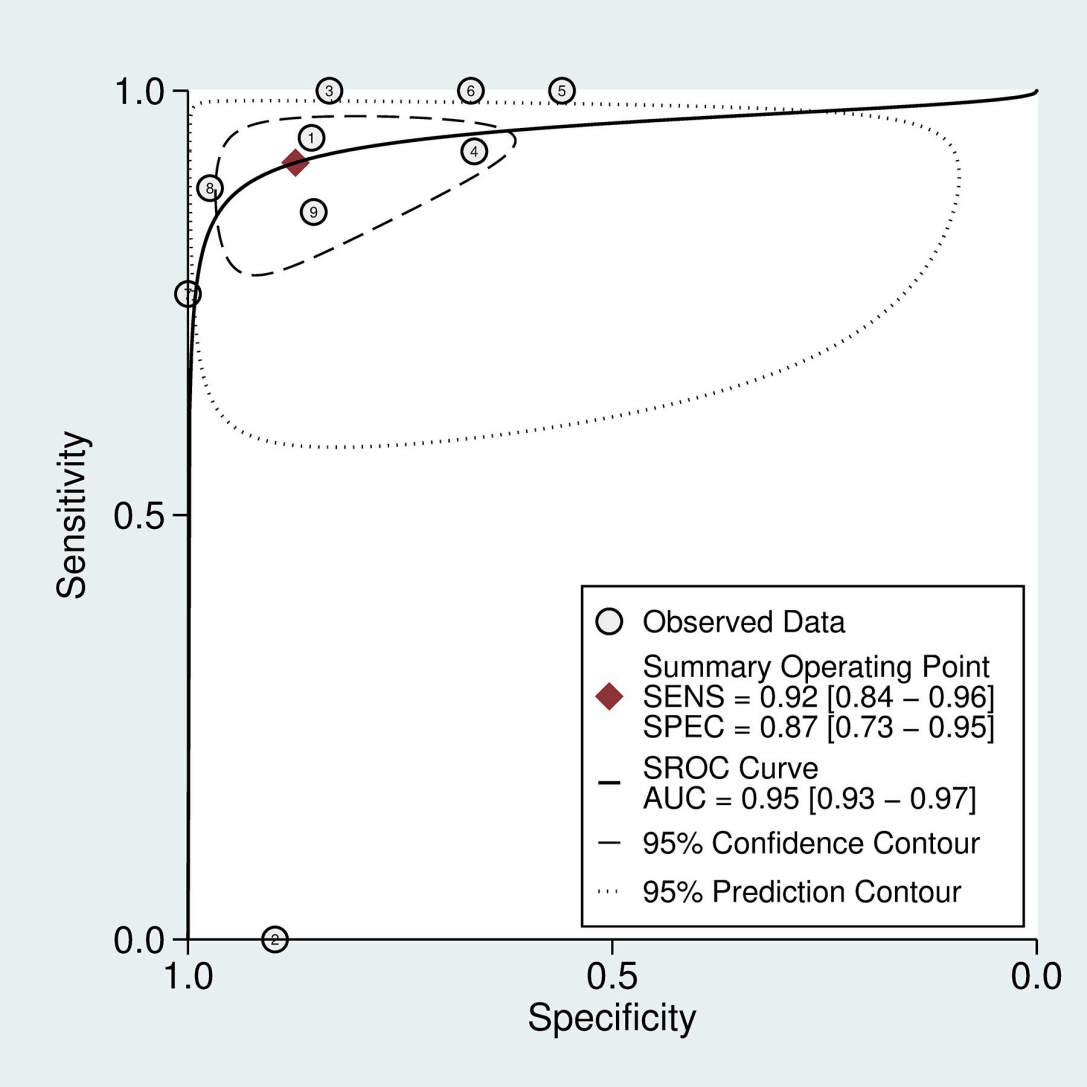
SROC curve for 20WBCT at detecting coagulopathy. SROC curves for 20WBCT at detecting severe coagulopathy defined as INR>5 or fibrinogen <50 mg/dL.
