# Supplementary material for: The 20-minute whole blood clotting test (20WBCT) for snakebite coagulopathy—A systematic review and meta-analysis of diagnostic test accuracy
Source: PLoS Negl Trop Dis. 2021 Aug 10;15(8):e0009657. doi: 10.1371/journal.pntd.0009657 (PMC8405032; doi:10.1371/journal.pntd.0009657)
Supplement: S1 Text — A full list of search strategies and results broken down by database. (DOCX) [file pntd.0009657.s001.docx]

**S1 Text - Systematic review search criteria**

## Search Strategies

Database: Medline (Ovid MEDLINE Epub Ahead of Print, In-Process & Other Non-Indexed Citations, Ovid MEDLINE Daily and Ovid MEDLINE) 1946 to present

Search Strategy:

--------------------------------------------------------------------------------

1 exp "Sensitivity and Specificity"/ or Snake Bites/di [Diagnosis] (573285)

2 (sensitivity or specificity).tw. (1010203)

3 ((pre-test or pretest) adj probability).tw. (2136)

4 post-test probability.tw. (566)

5 predictive value$.tw. (105046)

6 likelihood ratio$.tw. (15058)

7 diagnos*.tw. (2384710)

8 1 or 2 or 3 or 4 or 5 or 6 or 7 (3525410)

9 20WBCT.tw. (6)

10 20-WBCT.tw. (0)

11 WBCT20.tw. (8)

12 WBCT-20.tw. (3)

13 clotting*.tw. (22781)

14 Blood Coagulation/ (42700)

15 coagulopath*.tw. (14119)

16 coagulat*.tw. (97396)

17 9 or 10 or 11 or 12 or 13 or 14 or 15 or 16 (141294)

18 Snake Bites/ (4547)

19 exp Snake Venoms/ (18184)

20 exp Viperidae/ (4619)

21 snake*.tw. (20009)

22 envenom*.tw. (5102)

23 viper*.tw. (5038)

24 18 or 19 or 20 or 21 or 22 or 23 (35804)

25 8 and 17 and 24 (497)

Database: Embase 1974 to present

Search Strategy:

--------------------------------------------------------------------------------

1 exp "Sensitivity and Specificity"/ (347465)

2 (sensitivity or specificity).tw. (1272430)

3 ((pre-test or pretest) adj probability).tw. (3839)

4 post-test probability.tw. (818)

5 predictive value$.tw. (157017)

6 likelihood ratio$.tw. (20407)

7 diagnos*.tw. (3403859)

8 *Diagnostic Accuracy/ (11790)

9 snakebite/di [Diagnosis] (399)

10 1 or 2 or 3 or 4 or 5 or 6 or 7 or 8 or 9 (4532826)

11 20WBCT.tw. (7)

12 20-WBCT.tw. (2)

13 WBCT20.tw. (10)

14 WBCT-20.tw. (3)

15 clotting*.tw. (32322)

16 exp blood clotting/ (213917)

17 coagulopath*.tw. (22362)

18 coagulat*.tw. (132066)

19 11 or 12 or 13 or 14 or 15 or 16 or 17 or 18 (334621)

20 snakebite/ (5841)

21 exp snake venom/ (25955)

22 exp snake/ (13697)

23 snake*.tw. (21337)

24 envenom*.tw. (6313)

25 viper*.tw. (5954)

26 20 or 21 or 22 or 23 or 24 or 25 (46071)

27 10 and 19 and 26 (999)

Database: Global Health <1973 to 2020 Week 05>

Search Strategy:

--------------------------------------------------------------------------------

1 (sensitivity or specificity).tw. (138854)

2 ((pre-test or pretest) adj probability).tw. (159)

3 post-test probability.tw. (95)

4 predictive value$.tw. (15707)

5 likelihood ratio$.tw. (2276)

6 diagnos*.tw. (367674)

7 sensitivity analysis/ (1322)

8 exp diagnosis/ (172543)

9 1 or 2 or 3 or 4 or 5 or 6 or 7 or 8 (466132)

10 20WBCT.tw. (6)

11 20-WBCT.tw. (0)

12 WBCT20.tw. (5)

13 WBCT-20.tw. (2)

14 clotting*.tw. (5105)

15 blood coagulation/ (2423)

16 coagulopath*.tw. (1240)

17 coagulat*.tw. (10927)

18 10 or 11 or 12 or 13 or 14 or 15 or 16 or 17 (13152)

19 snake bites/ (2425)

20 snake venom/ (1539)

21 exp snakes/ (7481)

22 snake*.tw. (9402)

23 envenom*.tw. (6030)

24 viper*.tw. (4492)

25 19 or 20 or 21 or 22 or 23 or 24 (12436)

26 9 and 18 and 25 (162)

Scopus

*( ( TITLE-ABS-KEY ( sensitivity OR specificity OR diagnos* ) OR TITLE-ABS-KEY ( ( ( pre-test OR pretest ) W/3 probability ) ) OR TITLE-ABS-KEY ( "post-test probability" ) OR TITLE-ABS-KEY ( "predictive value*" ) OR TITLE-ABS-KEY ( "likelihood ratio*" ) ) ) AND ( ( TITLE-ABS-KEY ( 20wbct ) OR TITLE-ABS-KEY ( 20-wbct ) OR TITLE-ABS-KEY ( wbct20 ) OR TITLE-ABS-KEY ( wbct-20 ) OR TITLE-ABS-KEY ( clotting* ) OR TITLE-ABS-KEY ( coagulopath* ) OR TITLE-ABS-KEY ( coagulat* ) ) ) AND ( ( TITLE-ABS-KEY ( snake* OR envenom* OR viper* ) OR TITLE-ABS-KEY ( ( ( snake* OR viper* ) W/5 venom* ) ) ) )*

Web of Science Core Collection

#1 TOPIC: (sensitivity or specificity or diagnos*) OR TOPIC: (((pre-test or pretest) near/3 probability)) OR TOPIC: ("post-test probability") OR TOPIC: ("predictive value*") OR TOPIC: ("likelihood ratio*")

#2 TOPIC: (20WBCT) OR TOPIC: (20-WBCT) OR TOPIC: (WBCT20) OR TOPIC: (WBCT-20) OR TOPIC: (clotting*) OR TOPIC: (coagulopath*) OR TOPIC: (coagulat*)

#3 TOPIC: (snake*) OR TOPIC: (envenom*) OR TOPIC: (viper*) OR TOPIC: (((snake* OR viper*) near/5 venom*))

#4 #3 AND #2 AND #1

The WHO Global Index Medicus Regional Libraries <https://pesquisa.bvsalud.org/gim/?lang=en>

*(tw:(sensitivity or specificity or diagnos* or ((pre-test or pretest) and probability) or "post-test probability" or "predictive value*" or "likelihood ratio*")) AND (tw:(20WBCT or 20-WBCT or WBCT20 or WBCT-20 or clotting* or coagulopath* or coagulat*)) AND (tw:(snake* or envenom* or viper*))*

## Search Results

| Database | Results 10/02/2020 | Results 09/12/2020 |
| --- | --- | --- |
| Ovid Medline | 497 | 524 |
| Ovid Embase | 999 | 1072 |
| Ovid Global Health | 162 | 172 |
| Scopus | 1077 | 1099 |
| Web of Science Core Collection | 509 | 545 |
| WHO Global Index Medicus | 168 | 183 |
| TOTAL | 3412 | 3595 |
| Total after deduplication | 1948 | 2015 |
| Unique since 10/02/2020 |  | 109 |
