# Supplementary material for: The 20-minute whole blood clotting test (20WBCT) for snakebite coagulopathy—A systematic review and meta-analysis of diagnostic test accuracy
Source: PLoS Negl Trop Dis. 2021 Aug 10;15(8):e0009657. doi: 10.1371/journal.pntd.0009657 (PMC8405032; doi:10.1371/journal.pntd.0009657)
Supplement: Attachment — Submitted filename: 20WBCTSR_Response to reviewers.docx [file pntd.0009657.s002.docx]

Dr. Thomas lamb

Myanmar Oxford Clinical Research Unit

Centre of Tropical Medicine and Global Health

Nuffield Department of Medicine

University of Oxford

07 July 2021

Dear Professor Chippaux,

We thank you as deputy editor and the reviewers for taking time to provide critical feedback on the manuscript entitled,

*The 20-minute whole blood clotting test (20WBCT) for snakebite coagulopathy – a systematic review and meta-analysis of diagnostic test accuracy.*

Please find our replies (in red) to the reviewers’ comments (black) listed below.

Yours sincerely,

Dr. Thomas Lamb

Reviewer #4: Systematic review was said to include 15 studies. However, there was only one study from Nepal [Ref. 30]. In fact, data presented in table is not in accordance with the study published. Navin Bhatt is the first and corresponding author, while Samir Kumar Sharma the last one. Please, review reference in which Sharma was the first author in 2020.

Thank you for identifying this confusion. The unpublished datasets provided by N Bhatt and S Sharma were provided following communication to N Bhatt from the publication (31) entitled “Management of Pit Viper Envenoming without Antivenom: A Case Series”. We refer to the use of two published data sets from one original publication in the sentence on line 241-243 including a citation to the above paper. We have removed the citation to the original article from Table 1 to remove confusion.

Line 252 paragraph is incomplete.

Thank you for highlighting this error, this has now been corrected with a link to the data repository.

Correct typing for Thongtonyong, 2020 (ref. 32) in Table 1 and 2.

Thank you for highlighting this spelling error. This has now been corrected.

Reviewer #4: Coagulation tests are important in indicating the snakebite patients who should receive antivenom, and in monitoring the recovery of the coagulopathy. Oddly, this review showed that 20WBCT has been performed after antivenom treatment in the minority of the studies, although most of the studies were specifically designed to assess the validity of the test. Please, discuss this apparent inconsistence.

We agree that despite widespread use of the 20WBCT for assessing antivenom response, it is concerning that few studies aimed to assess the diagnostic test accuracy of the 20WBCT for this purpose. This has been outlined on line 425-427 and suggestions for further work on this area are discussed in the discussion lines 427-429 and lines 453-455 and conclusion lines 482-484.

Would 20WBCT be more effective to detect a “all or nothing” coagulation disturbance, as originally described by Warrell, and less efficient to detect partial coagulation abnormality, as expected few hours after antivenom administration?

We thank the reviewer for this comment. The authors agree that the 20WBCT appears to perform better at detecting severe coagulopathy. This is supported by figures 4a and 4b. We have added a sentence to the discussion (line 390-392) underlining this finding.


Reviewer #1: Line 173: “Although subtle variations in methodology have been used, the most common method, as described by the WHO, has been implemented in national snakebite guidelines” : is this a general statement or a statement specific to Myanmar?

We apologise for the confusion in the original manuscript, we have now clarified this statement so that it reads,

“Although subtle variations in methodology have been used, the most common method, as described by the WHO, has been implemented in national snakebite guidelines, such as Myanmar’.

Line 252: incomplete

Thank you for highlighting this error, this has now been corrected with a link to the data repository.

Page 15: Dsilva – please correct the spelling of Daboia

Thank you for highlighting this spelling error, this has now been corrected.

Fig 4b: The unit of fibrinogen value must be indicated on the x-axis label
This has now been corrected on the revised draft.

Line 368: g/dL or mg/dL?

Thank you for highlighting this error, this has now been corrected.


Reviewer #1:

Although the authors have compared the outcome of WBCT20 with the INR and fibrinogen measurements taken at the same time point, across different studies, the time from the snake bite to the test has not adequately appreciated in analysis. The utility of WBCT20 depends not only on the performance of the test in effectively detecting various severities of VICC. The diagnostic utility of WBCT20 may also depend on how early it detects VICC. This aspect is cannot-be adequately addressed by sub-group analysis of on-admission (admission times could variry greatly across settings) and post-antivenom.

WBCT20 This may be very important in envenomings by certain snake species that often leads to the gradual transformation of mild VICC to severe VICC. For example, if a patient bitten by Russell’s viper presents to a hospital two hours after the bite and the WBCT20 becomes negative at an INR of 2.0 (so that the antivenom is not indicated) but six hours later WBCT20 becomes positive at an INR of >10, the diagnostic utility of the test is quite low. Therefore, I suggest the authors address the above issue and include a column to table 1 showing the median and IQR/ range of the time from the bite to test in individual studies, if they have presented the data.

We thank the reviewer for identifying this key observation. We agree that prior antivenom therapy and time from bite to 20WBCT are key confounders that may affect the diagnostic accuracy of the 20WBCT. Unfortunately, few of the studies (3/15) reported median time to 20WBCT from bite.

We have added this potential confounder to Table 1 and have expanded this discussion as a limitation of the study finding on line 470-473 that reads,

“Time from bite to index test is another probable confounding factor when comparing diagnostic test accuracy across studies. Unfortunately, just three studies in this meta-analysis reported the median time from bite to index test, precluding a bivariate regression analysis to assess the impact of heterogeneity.”

In addition, if it is possible, a subgroup analysis for <4hr and >4hr from the bite or even <6hr and >6hr would be quite important.

Thank you for this suggestion. Unfortunately, the incomplete data and lack of individual patient data reporting time to index test precludes this analysis.

Reviewer #2:
1.      There are different mechanisms of coagulopathy among snake families. For example, pit vipers mainly consume fibrinogen, while true vipers also affect other clotting factors. I wonder if there are differences in 20WBCT performances for pit vipers vs. other snakes.

Thank you for highlighting this interesting observation. We agree that the diverse effects of venom are likely to result in varying degree of clotting abnormality and in turn, affect the diagnostic accuracy of the 20WBCT. Unfortunately, the data included in this review infrequently identified snake down to species level and includes too many confounders to tease out the specific impact on 20WBCT test accuracy between different snake genera. We expanded upon this limitation with the following statement on lines 468-470.

“It is likely that the 20WBCT diagnostic test accuracy in snakebite envenoming varies for bites from different snake genera.”

2.      Line 171: ‘poisoning’ suggests ingestion of toxins. For snakebite, envenoming may be more appropriate.

We agree that term ‘poisoning’ is misleading and open to misinterpretation, however as this is a direct quote from the original paper by Warrell et al, we have decided to leave it unchanged in quotation marks.
